# Scalable Culture Strategies for the Expansion of Patient-Derived Cancer Stem Cell Lines

**DOI:** 10.1155/2019/8347595

**Published:** 2019-02-06

**Authors:** Ana Teresa Serra, Margarida Serra, Ana Carina Silva, Tamara Brckalo, Anita Seshire, Catarina Brito, Michael Wolf, Paula M. Alves

**Affiliations:** ^1^Instituto de Tecnologia Química e Biológica António Xavier, Universidade Nova de Lisboa, Av. da República, 2780-157 Oeiras, Portugal; ^2^Instituto de Biologia Experimental e Tecnológica (iBET), Apartado 12, 2780-901 Oeiras, Portugal; ^3^Merck KGaA, Merck Serono, ImmunoOncology, Emerging Immunotherapies, Frankfurter Str. 250, 64293 Darmstadt, Germany; ^4^Merck KGaA, Biopharma, Global R&D, Translational Innovation Platform Oncology, Cellular Pharmacology, Frankfurter Str. 250, 64293 Darmstadt, Germany

## Abstract

Cancer stem cells (CSCs) have recently raised great interest as a promising biological system for designing effective cancer therapies. The scarcity of CSCs *in vivo* and the consequent low numbers obtained from biopsies represent a major hurdle to the development of such strategies. It is therefore necessary to design robust scalable methods to enable efficient expansion of bona fide CSCs *in vitro*. Here, we evaluated the applicability of computer-controlled bioreactors combined with 3D aggregate culture and microcarrier technology, widely used in stem cell bioprocessing, for the expansion and enrichment of CSCs isolated from different types of solid tumors—colorectal cancer (CRC) and non-small-cell lung cancer (NSCLC) from two patients. Results show that these culture strategies improved cell expansion and CSC enrichment. Both patient-derived CSC lines were able to grow on microcarriers, the best results being achieved for PPlus 102-L, Pro-F 102-L, Fact 102-L, and CGEN 102-L beads (5-fold and 40-fold increase in total cell concentration for CRC and NSCLC cells, respectively, in 6 days). As for 3D aggregate culture strategy, the cell proliferation profile was donor dependent. NSCLC cells were the only cells able to form aggregates and proliferate, and the flat-bottom bioreactor vessel equipped with a trapezoid-shaped paddle impeller was the most efficient configuration for cell growth (21-fold increase in cell concentration achieved in 8 days). Serum-free medium promotes CSC enrichment in both 3D aggregate and microcarrier cultures. The protocols developed herein for CSC expansion have the potential to be transferred to clinical and industrial settings, providing key insights to guide bioprocess design towards the production of enriched CSC cultures in higher quantity and improved quality.

## 1. Introduction

Cancer stem cells (CSCs) represent a promising target for effective anticancer therapies [1, 2] as these immortal tumor-initiating cells have the capacity to self-renew and differentiate into the spectrum of cell types observed in tumors [3–5]. Due to their characteristics (enhanced motility, invasion, tumor-initiating ability, and resistance to chemotherapy), CSCs are thought to be the basis for tumor initiation, development, metastasis, and recurrence, thus contributing to the failure of conventional cancer treatments [6, 7].

It has been reported that CSCs exist within almost every solid tumor [5, 6] at a very small number (<0.04%) [4]. Difficulties in identifying these cells, their reduced number, and the lack of protocols for efficient CSC expansion and enrichment have hindered the development of effective CSC-targeted therapies. The use of stirred culture systems, previously applied to the (i) expansion and differentiation of human stem cells [8–11] and ii) cultivation of (primary) cancer cells [12–14], can offer great advantages for CSC expansion over static culture systems [15–18], including higher cell production yields, reproducibility, scalability, and easy transfer to clinic and industry [19].

In this work, computer-controlled stirred tank bioreactors combined with 3D cell aggregate cultures as well as microcarrier technology were applied for the first time to expand and enrich CSCs from two different patient-derived cell lines—non-small-cell lung cancer (NSCLC) and colorectal cancer (CRC). The findings reported herein provide novel knowledge to guide cell bioprocess design towards the production of CSC in higher quantity and improved quality, which are key requisites for their application in drug discovery and in the development of new cancer therapeutics.

## 2. Material and Methods

### 2.1. Cell Source

CSC lines were established in Merck Biopharma, ImmunoOncology, following a proprietary protocol. Tumor cells were derived from lung and colorectal cancer patients and purchased from Indivumed (Hamburg, Germany). Classification of the tumors was large-cell carcinoma, NOS, and colorectal carcinoma. CSC lines (CRC and NSCLC) were routinely propagated in collagen I-coated T-flasks as described in supplemental online data.

### 2.2. Culture of CSC Lines as Aggregates in Stirred Tank Bioreactors

CSC lines were inoculated as single cells in computer-controlled stirred tank bioreactors at a concentration of 0.25 × 10^6^ cell/mL and cultured during 8 days in two different bioreactor configurations—round-bottom bioreactor vessel equipped with a pitched 4-bladde impeller (BR-R/P4b) and flat-bottom bioreactor vessel equipped with a trapezoid-shaped paddle impeller (BR-F/T) (DASGIP CellFerm-Pro bioreactor system, Eppendorf AG). Two culture medium formulations (serum-containing medium (SCM) and serum-free medium (SFM)) and four cell aggregate dissociation protocols were tested (more information available in the supplemental online data).

### 2.3. Culture of CSCs on Microcarriers

CSC lines were inoculated as single cells with empty microcarriers (2.0 × 10^4^ cell/cm^2^) in ultra-low-attachment plates and cultured for 6 days at 37°C in a humidified atmosphere of 5% CO_2_ using SCM or SFM. Eight commercially available microcarriers were tested: Cytodex1™, Cytodex3™, PPlus 102-L, Pro-F 102-L, Fact 102-L, CGEN 102-L, Cytopore2™, and CultiSpher®-S as described in the supplemental online data.

### 2.4. Analytical Methods

Protocols for CSC characterization are included in the supplemental online data.

## 3. Results and Discussion

Primary CSC lines generated from colorectal cancer (CRC) and non-small-cell lung cancer (NSCLC) from two patients, when routinely cultured in static adherent culture systems, show percentage of ALDH^+^ cells higher than 50% and have the capacity to generate tumor spheres (SFUs) (data not shown). For their expansion, different bioreactor configurations and aggregate dissociation protocols were evaluated. In addition, microcarrier-based cultures were investigated; eight different microcarriers (selection made based on previous works performed with human stem cells [10, 11], supplemental online Table S1) were screened for CRC and NSCLC cell expansion. The impact of culture medium composition (serum-containing medium (SCM) and serum-free medium (SFM)) on the cell expansion ratio and CSC enrichment was also evaluated.

**Figure 1:**
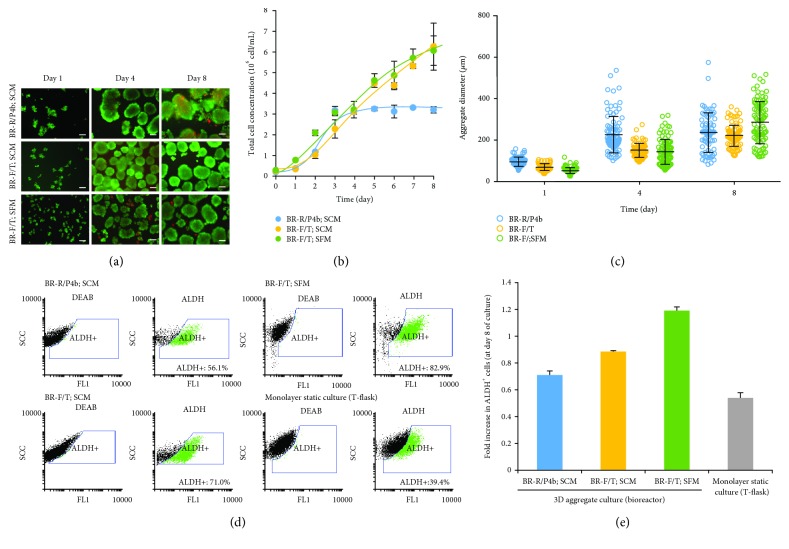
Effect of bioreactor configuration and culture medium composition on the expansion of NSCLC cells. Cells were inoculated at 0.25 × 10^6^ cells/mL and cultured in a round-bottom bioreactor vessel equipped with a pitched 4-bladde impeller (BR-R/P4b) or in a flat-bottom bioreactor vessel equipped with a trapezoid-shaped paddle impeller (BR-F/T) using serum-containing medium (SCM) and serum-free medium (SFM). (a) Fluorescence microscopy images of NSCLC cultures at days 1, 4, and 8 of the three bioreactor experiments. Viability analysis of cultures stained with fluorescein diacetate (FDA—live cells, green) and propidium iodide (PI—dead cells, red). Scale bars: 100 *μ*m. (b) Growth curve expressed in terms of cell number per volume of medium (determined by crystal violet nuclei stain assay; error bars denote SD of 3 measurements). (c) Aggregate size (average diameters of aggregates were determined by ImageJ software; error bars denote SD of measurements from 100 aggregates). (d) Flow cytometry analysis of NSCLC culture in bioreactors and in monolayer static systems: percentage of ALDH^+^ cells at day 8 of culture. The left panel shows the dot blot of ALDEFLUOR™ assay with an inhibitor (DEAB), and the right panel shows the dot blot without an inhibitor. The ALDH^+^ cell population is identified in green. (e) Fold increase in ALDH^+^ cells obtained at day 8 of culture in bioreactors and monolayer static culture systems in relation to the inoculum population.

NSCLC cells were able to grow as aggregates in computer-controlled stirred tank bioreactors independently of the medium used (SCM or SFM) (Figures 1(a) and 1(b)). Importantly, cell growth kinetics (Figure 1(b)) and aggregate size (Figure 1(c)) seem to be driven by hydrodynamics, evaluated through the use of different vessel designs and impeller geometries (see Material and Methods). BR-F/T was the most efficient configuration as it allowed (i) cell growth as spherical aggregates of uniform size (Figures 1(a) and 1(c)) and (ii) a 21-fold increase in cell concentration after day 8 (Figure 1(b)). The maximum cell concentration (Figure 1(b)) and cell expansion factors achieved in our study are within the range of values reported by our group [10] and others [20–22] for the expansion of human pluripotent stem cells as 3D aggregates in bioreactors. The small variations observed in the cell growth profile may reflect the distinct cell types and the different culture conditions used in those studies, such as the culture medium formulation and feeding strategy. Lower cell concentrations were obtained in BR-R/P4b configuration; the cell growth arrest observed for this bioreactor at day 4 might be related to oxygen diffusion limitations within the aggregate as suggested by the high heterogeneity and size of aggregates analyzed (Figure 1(c)), and the values reported to exhibit hypoxia regions (diameter > 400 *μ*m) [23]. Noteworthy, aggregates cultured in BR-F/T presented the highest percentage of ALDH^+^ cells (>70%) (Figure 1(d)). Culture medium composition impacts on CSC enrichment. Indeed, a 1.2-fold increase in ALDH^+^ cells relative to inoculum was observed in SFM (BR-F/T; SFM), contrasting to SCM cultures where no increase was attained (BR-F/T; SCM) (Figure 1(e)).

**Figure 2:**
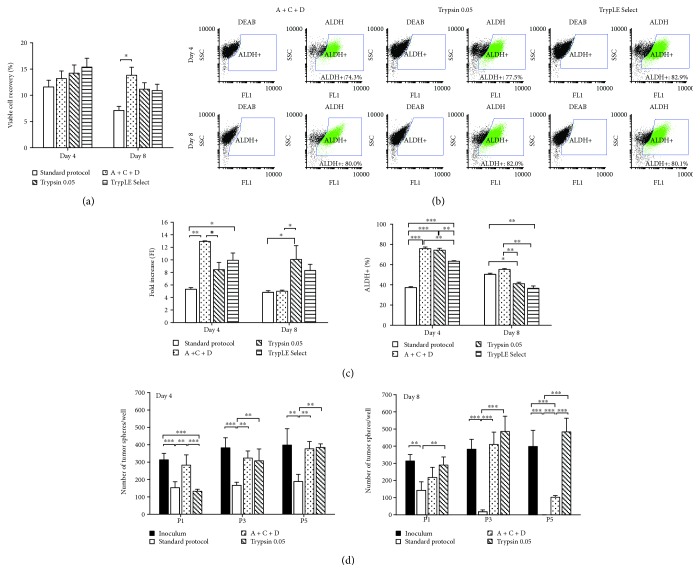
Results from harvesting studies of NSCLC cell aggregates cultured in stirred tank bioreactors. Cells were cultured in BR-F/T using serum-free medium (SFM) and harvested at days 4 and 8 of culture using different cell dissociation protocols, namely, Standard protocol, Accutase+Collagenase III+DNase I (A+C+D), Trypsin 0.05%, and TrypLE Select. (a) Percentage of viable cells recovered after each dissociation protocol. Values were estimated by the *X*_t_/*X*_ti_ ratio, where *X*_t_ is the number of total cells recovered after dissociation protocol (determined by trypan blue exclusion assay) and *X*_ti_ is the number of total cells harvested from the bioreactor (determined by crystal violet nucleic stain assay) (error bars denote SD of 2 measurements). (b) Flow cytometry analysis of NSCLC culture after cell aggregate dissociation by A+C+D, trypsin 0.05, and TrypLE Select protocols: percentage of ALDH^+^ cells recovered at days 4 and 8 of culture. The left panel shows the dot blot of ALDEFLUOR™ assay with an inhibitor (DEAB), and the right panel shows the dot blot without an inhibitor. The ALDH^+^ cell population is identified in green. (c) Readhesion and expansion capacity of NSCLC cells harvested at days 4 and 8 using different dissociation protocols. Cells were dissociated and plated in collagen I-coated flasks and cultured for 4 days in static culture conditions using serum-containing medium. Left panel: fold increase in total cell concentration (estimated by crystal violet nucleic stain assay; error bars denote SD of 2 measurements). Right panel: percentage of ALDH^+^ cells determined after 4 days of culture (error bars denote SD of 2 measurements). (d) Number of tumor spheres per well generated by NSCLC cells derived from the inoculum and harvested from bioreactor culture at days 4 (left panel) and 8 (right panel) using different dissociation protocols. The number of tumor spheres was estimated up to five passages. Error bars denote SD of measurements from 4 wells. Statistical significance is indicated as follows: ^∗^ *p* < 0.05, ^∗∗^ *p* < 0.01, and ^∗∗∗^ *p* < 0.001.

Aggregates were harvested at two time points, days 4 and 8, and the impact of different aggregate dissociation protocols on the ability of NSCLC cells to readhere to collagen I-coated flasks and to form tumor spheres was investigated (Figure 2). Results indicate that recovery yields of viable cells (7-15%) (Figure 2(a)) and ALDH^+^ subpopulations (74-83%) (Figures 1(d) and 2(b)) are independent of the protocol used. Nonetheless, A+C+D and Trypsin 0.05 were the enzymatic solutions best suited for dissociation of NSCLC cell aggregates. Both protocols improved cell readhesion and expansion capacity (up to 2-fold) and enhanced cells' ability to generate tumor spheres when compared to the standard protocol for the two harvesting days (Figures 2(c) and 2(d)). In contrast, TrypLE Select showed lower percentage of ALDH^+^ subpopulation than A+C+D and Trypsin 0.05 (Figure 2(c)), and the ability to generate tumor spheres was negligible (data not shown). The evaluation of other digestion reagents such as those recently reported for the isolation of human glioma stem cells [24] may be considered in the future to improve cell recovery yields from NSCLC cell aggregates.

**Figure 3:**
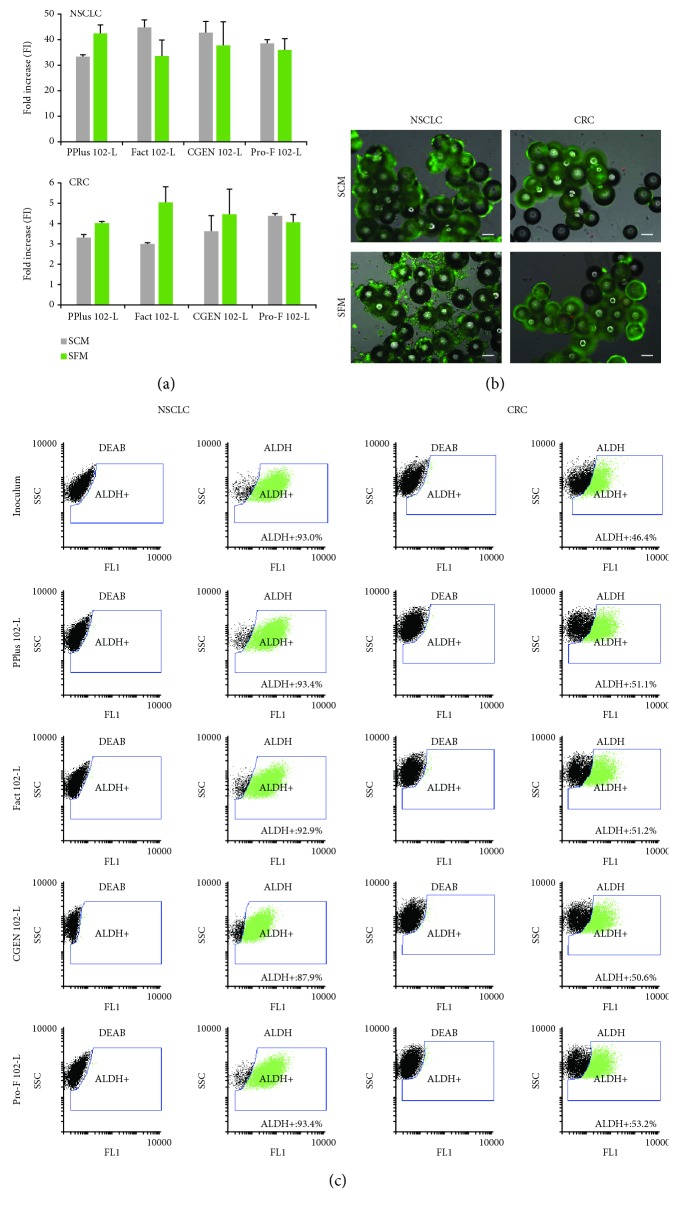
NSCLC and CRC cell culture in four different microcarriers: PPlus 102-L, Pro-F 102-L, Fact 102-L, and CGEN 102-L. Cells were inoculated at 0.2 × 10^4^ cell/cm^2^ and cultured for 6 days under static culture systems using two different culture media: serum-containing medium (SCM) and serum-free medium (SFM). (a) Fold increase in NSCLC (upper panel) and CRC (lower panel) cell concentration at day 6 of culture on microcarriers using both culture media. Total cell concentration was determined by crystal violet nucleic stain assay. (b) Phase-contrast and fluorescence microscopy images of NSCLC and CRC cells cultured on PPlus 102-L microcarriers. Viability analysis of cultures stained with fluorescein diacetate (FDA—live cells, green) and propidium iodide (PI—dead cells, red). Scale bars: 100 *μ*m. (c) Flow cytometry analysis of NSCLC and CRC cell population at inoculum and after 6 days of culture in microcarriers using serum-free medium. The left panel shows the dot blot of ALDEFLUOR™ assay with an inhibitor (DEAB), and the right panel shows the dot blot without an inhibitor. The ALDH^+^ cell population is identified in green.

When NSCLC cells were cultured in microcarriers, higher expansion ratios (up to 48) were obtained in less culture time (6 days) when compared to aggregate culture in bioreactors, the exception being Cytopore2™ beads (Table 1). In addition, cells efficiently attach and grow on microcarriers using both SCM and SFM (Figures 3(a) and 3(b)). The use of SFM did not compromise ALDH activity of NSCLC cells (percentage of ALDH^+^ cells obtained at day 6 is similar to that of the inoculum) (Figure 3(c)).

Microcarrier-based culture was also suitable for the expansion of CRC cells, and the highest increase in cell concentration (>3-fold) was observed for PPlus 102-L, Fact 102-L, CGEN 102, and Pro-F 102-L beads (Table 1). The culture medium seems to have a negligible impact on the expansion ratio and microcarrier colonization (Figures 3(a) and 3(b)). In particular, higher percentages of ALDH^+^ subpopulations in relation to the inoculum were observed for cultures using SFM (Figure 3(c)). The two macroporous microcarriers evaluated (CultiSpher®-S and Cytopore2™) did not support CRC cell expansion. Although initial cell attachment to the bead surface was observed, cell proliferation inside microcarriers did not occur (supplemental online Figure S1, Table 1). In addition, these patient-derived CSC lines did not proliferate when cultured as aggregates in computer-controlled stirred tank bioreactors, showing low aggregation and expansion capacity regardless of the different media and inoculum concentrations (0.1, 0.25, and 0.4 × 10^6^ cells/mL) tested (supplemental online Figure S2). The differences in aggregation and growth observed between NSCLC and CRC cells may be related to the distinct sources (tissues and patients) from which the cells were derived. Alternative/complementary approaches (e.g., cell microencapsulation in hydrogels as reported for human cancer cell lines [13, 14]) might be considered in the future for the scalable expansion of CRC cells.

## 4. Conclusion

This work describes, for the first time, the successful application of computer-controlled stirred tank bioreactors combined with 3D aggregate cultures as well as microcarrier technology to expand and enrich human CSCs. Despite the fact that there is no universal culture strategy capable of embracing different types/patient-derived CSCs, the protocols developed herein for CSC expansion can be easily screened prior to their transfer to clinical and industrial settings. This study also provides key insights to guide bioprocess design towards scalable production of patient-derived CSCs with improved quality. This will potentiate their application in drug discovery and for the development of new cancer therapeutics.

## Figures and Tables

**Table 1:** Effect of the microcarrier type on CRC (colorectal cancer) and NSCLC (non-small-cell lung cancer) cell growth using serum-containing medium.

Microcarrier type	Cytodex1™	PPlus 102-L	Fact 102-L	Cytodex3™	CGEN 102-L	Pro-F 102-L	Cytopore2™	CultiSpher®-S
*CSC line*	CRC							
*X*_inoc_ (×10^4^ cell/cm^2^)	2.0							
*X*_6d_ (×10^4^ cell/cm^2^)	2.9 ± 0.5	6.6 ± 0.3	6.0 ± 0.1	2.9 ± 0.4	7.3 ± 1.5	8.8 ± 0.2	1.9 ± 0.1	1.5 ± 0.2
Expansion ratio^∗^	1.4 ± 0.3	3.3 ± 0.1	3.0 ± 0.1	1.5 ± 0.2	3.6 ± 0.8	4.4 ± 0.1	1.0 ± 0.1	0.8 ± 0.1
*CSC line*	NSCLC							
*X*_inoc_ (×10^4^ cell/cm^2^)	2.0							
*X*_6d_ (×10^4^ cell/cm^2^)	95.8 ± 19.2	66.7 ± 1.4	89.6 ± 5.9	62.0 ± 18.4	85.4 ± 8.8	77.1 ± 3.0	31.8 ± 0.7	66.1 ± 0.7
Expansion ratio^∗^	47.9 ± 9.6	33.3 ± 0.7	44.8 ± 2.9	31.0 ± 9.2	42.7 ± 4.4	38.5 ± 1.5	15.9 ± 0.4	33.1 ± 0.4

^∗^Fold increase in total cell concentration attained at day 6 of culture determined by the ratio between cell concentration achieved at day 6 (*X*_6d_) and cell concentration used at inoculum (*X*_inoc_), respectively.

## Data Availability

No data were used to support this study.

## Supplementary Materials

Supplementary Materials Figure S1: Phase-contrast and fluorescence microscopy images of CRC cells cultured on macroporous microcarriers, Cytopore2™ and CultiSpher®-S, at days 1, 4, and 6. Viability analysis of cultures stained with fluorescein diacetate (FDA—live cells, green) and propidium iodide (PI—dead cells, red). Scale bars: 100 *μ*m. Figure S2: Fluorescence microscopy images of CRC cultures at day 4 on BR-F/T using culture medium with serum (a) and serum-free medium (b). Viability analysis of cultures stained with fluorescein diacetate (FDA—live cells, green) and propidium iodide (PI—dead cells, red). Scale bars: 100 *μ*m. Figure S3: Representative image of the ImageJ mask for aggregate size distribution analysis. Table S1: Main characteristics of microcarriers tested. Classification of microcarrier type was indicated according to Chen et al. [11]. ECM: extracellular matrix.
