# Supplementary material for: Scalable Culture Strategies for the Expansion of Patient-Derived Cancer Stem Cell Lines
Source: Stem Cells Int. 2019 Feb 6;2019:8347595. doi: 10.1155/2019/8347595 (PMC6409046; doi:10.1155/2019/8347595)
Supplement: Supplementary Materials — Figure S1: Phase-contrast and fluorescence microscopy images of CRC cells cultured on macroporous microcarriers, Cytopore2™ and CultiSpher®-S, at days 1, 4, and 6. Viability analysis of cultures stained with fluorescein diacetate (FDA—live cells, green) and propidium iodide (PI—dead cells, red). Scale bars: 100 μm. Figure S2: Fluorescence microscopy images of CRC cultures at day 4 on BR-F/T using culture medium with serum (a) and serum-free medium (b). Viability analysis of cultures stained with fluorescein diacetate (FDA—live cells, green) and propidium iodide (PI—dead cells, red). Scale bars: 100 μm. Figure S3: Representative image of the ImageJ mask for aggregate size distribution analysis. Table S1: Main characteristics of microcarriers tested. Classification of microcarrier type was indicated according to Chen et al. [11]. ECM: extracellular matrix. [file 8347595.f1.docx]

**Supplemental Online Data- Serra et al.**

**1. Supplemental Material and Methods:**

- 1. **Cell source and culture in adherent static culture systems**

CSC lines were established in Merck Biopharma, Immunooncology, following a proprietary protocol. Tumor cells were derived from a lung and colorectal cancer patients and purchased from Indivumed (Hamburg, Germany). Classification of the tumors was large cell carcinoma, NOS, and colorectal carcinoma. Indivumed holds informed consents of both patients. Tests for mycoplasma, bacteria and fungi, for HIV, hepatitis B and hepatitis C were negative. Cells are of epithelial like origin.

CSC lines (CRC and NSCLC) were routinely propagated in collagen I coated T-flasks (Corning) using DMEM/F12 medium supplemented with 2mM of L-Glutamine , 5% (v/v) of fetal bovine serum, 5 µg/mL of transferrin, 20 µg/mL of insulin, 1% (v/v) of penicillin/streptomycin, 20 µg/mL of gentamycin-sulfate and amphotericin B (all from PAN Biotech) and 12.5 µg/mL of bovine fetuin (Calbiochem). Cells were inoculated at 2.0×10^4^ cell/cm^2^, cultured for 4 days at 37^o^C in a humidified atmosphere of 5% CO_2_, and split using a stepwise procedure with accutase (PAA Laboratories GmbH) followed by collagenase III (Worthington Biochem. Corp) plus DNase I (Applichem) solution and mechanical disruption through a 30 μm mesh (pre-separation filter, Miltenyi Biotech).

- 1. **Culture of CSCs as aggregates**

**1.2.1. Culture of CSCs aggregates in stirred tank bioreactors**

CSCs were inoculated as single cells in stirred-tank bioreactors at a concentration of 0.25 ×10^6^cell/mL, in 200 mL of culture medium. Two different bioreactor configurations were evaluated namely i) round bottom bioreactor vessel equipped with pitched 4-bladde impeller (BR-R/P4b) and ii) flat bottom bioreactor vessel equipped with trapezoid shaped paddle impeller (BR-F/T) (DasGip cellferm-pro bioreactor system, Eppendorf AG). Cells were cultivated under defined and controlled culture conditions (CO_2_: 5 %; temperature: 37 °C; pO_2_: 20 % O_2_; surface aeration rate: 0.1 vvm; agitation rate: 70-110 rpm for BR-F/T and 70-120rpm for BR-R/P4B) during 8 days. Data acquisition and process control were performed using DASGIP® Control Software 4.0 (Eppendorf AG). Two different culture media were used: serum containing medium (described in section 1.1) and serum free medium composed by DMEM/F12 medium supplemented with 2 mM of L-Glutamine 5 µg/mL of transferrin, 20 µg/mL of insulin, 1% (v/v) of penicillin/streptomycin, 20 µg/mL of gentamycin-sulfate and amphotericin B (all from PAN Biotech), 12.5 µg/mL of bovine fetuin (Calbiochem), 20 ng/mL of EGF and 10 ng/mL of bFGF (R&D Systems), 4µg/mL of heparin (Stem Cell Technologies), 2% (v/v) of B27 supplement (Invitrogen), 0.006% (w/v) of glucose (Merck), 0.96 µg/mL of putrescine, 0.50 ng/mL of sodium selenite and 0.63 ng/mL of progesterone; all from Sigma. Medium exchange (50% replacement) was carried out at days 4 and 6 for the SCM experiments or daily from day 3 onwards for the SFM cultures, in order to avoid depletion of nutrients (e.g. glucose) and/or accumulation of toxic metabolites (e.g. lactate).

All aggregate cultures were monitored in terms of morphological analysis, cell concentration (nuclei and viable cell counting) and aggregate diameter distribution, as described below.

**1.2.2. Cell dissociation protocols**

At days 4 and 8 of bioreactor culture, NSCLC aggregates were harvested and dissociated to single cells using four different protocols. For all methods, aggregates were centrifuged (240x *g*, 6 min) and washed with DPBS and the same starting cell concentration was used. All incubation steps were performed at 37^o^C, unless otherwise stated. In the standard protocol, aggregate dissociation was performed using a stepwise procedure with accutase (PAA Laboratories GmbH) followed by collagenase III (Worthington Biochem. Corp) plus DNase I (Applichem) solution. Briefly aggregates were incubated with accutase for 5 minutes and after inactivation with HBSS supplemented with 2% (w/v) BSA , 1% (v/v) of penicillin/streptomycin, 20 µg/mL of gentamycin-sulfate and 500ng/mL of amphotericin B (hereafter designated as enzymatic inactivation solution), cells were washed twice with DPBS and incubated with collagenase solution (Collagenase III (200U/mL) + DNAse (100U/mL) + Antibiotics (1% (v/v) penicillin/streptomycin, 500ng/mL gentamycin, 20μg/mL amphotericin) during 14 minutes. Cells/clusters were pressed through a 30 µM Milteny pre-separation filter into a fresh tube, placed on ice, containing enzymatic inactivation solution. In the other dissociation protocols, cell aggregates were disrupted using 3 different enzymatic solutions: a solution of accutase supplemented with Collagenase III (200 U/mL) and DNase I (100 U/mL), Trypsin-EDTA (0.05%) (Gibco) and TrypLE Select (1x) (Gibco). For all these methods a similar protocol was used: cell suspension was incubated with the enzymatic solution for 10 minutes followed by mechanical disruption through a 30 µM Milteny pre-separation filter into a fresh tube, placed on ice, containing enzymatic inactivation solution.

**1.3. Culture of CSCs on Microcarriers**

CSCs were inoculated (2.0 x 10^4^ cell/cm^2^) with empty microcarriers in ultra-low-attachment 24-well plates (Corning Inc.) in 1 mL of culture medium (SCM or SFM) per well. Eight different commercially available microcarriers were tested: Cytodex 1™ (GE Healthcare), P Plus 102-L (Thermo Scientific HyClone), Fact 102-L ((Thermo Scientific HyClone), Cytodex 3™(GE Healthcare), CGEN 102-L (Thermo Scientific HyClone), Pro-F 102-L (Thermo Scientific HyClone), Cytopore2™ (GE Healthcare) and CultiSpher®-S (Percell) (Table S1). All microcarriers were prepared according to the manufacturer’s instructions and the amount of microcarriers used in each well was estimated to yield 3 cm^2^ of available area: Cytodex 1™ - 0.7 mg, P Plus 102-L- 8.3 mg, Fact 102-L- 8.3 mg, Cytodex 3™- 1.1 mg, CGEN 102-L- 8.3 mg, Pro-F 102-L- 8.3 mg, Cytopore2™ - 0.3 mg, CultiSpher®-S- 1 mg. Since there is no information regarding the value of surface area available per Cultisphere S bead or per gram, the concentration of these microcarriers was selected based on manufacture’s recommendations and our previous experience with other (stem) cell type. The cells were placed inside humidified incubators (37^o^C, 5% CO_2_) and cultured for up to 6 days in static conditions; medium was partially (50%) replaced at day 4. All cultures were monitored daily by microscopic inspection. Cell proliferation within 6 days of culture was determined using crystal violet nuclei staining assay (section 1.4.3). Cells were detached from microcarriers for ALDH activity determination using the cell dissociation protocol described in section 1.1.

**1.4. Analytical methods**

**1.4.1. Aggregate concentration and size distribution**

The concentration of aggregates was determined in 4-10 wells of a 96-well plate containing 1 mL aliquots of bioreactor culture samples using an inverted microscope (DMI6000, Leica). Their average diameters were measured by adjusting the threshold until the border of each aggregate, which were quantified using the Ferret diameter measurement algorithm from ImageJ open source software (Rasband, WS, ImageJ, U. S. National Institutes of Health, Bethesda, MD, USA, http://imagej.nih.gov/ij/, 1997–2012). A representative image of the ImageJ mask for aggregate size distribution analysis is presented in Figure S3.

**1.4.2. Assessing cell viability**

Cell membrane integrity assessment: cell aggregates and cell-microcarrier suspension samples were incubated with 20 µg/mL fluorescein diacetate (FDA; Sigma-Aldrich) and 10 µg/mL propidium iodide (PI; Sigma-Aldrich) in DPBS for 2-5 min. Samples were then observed under a fluorescence microscope (Leica Microsystems GmbH) and photographed using a Leica DFC 360 FX digital camera.

**1.4.3 Assessing total and viable cell concentration**

Nuclei staining: total cell numbers were assessed by crystal violet nuclei staining. Briefly, cell aggregates or cell attached on microcarriers were resuspended in lysis buffer (1% (w/v) Triton in 0.1 M citric acid (Merck)), vortexed and incubated at 37^o^C for at least 2 hours. Nuclei were stained with crystal violet dye (Merck) (0.1% (v/v) in lysis buffer) and the total number of cells counted in a Fuchs-Rosenthal haemocytometer chamber. Trypan Blue exclusion method: Cell concentration was estimated by counting cells using a Fuchs–Rosenthal haemocytometer. Viable cells were determined by using the trypan blue dye (Invitrogen) (0.1% (v/v) in DPBS) exclusion method.

**1.4.4. Re-adhesion and expansion capacity of CSC expanded in bioreactors**

NSCLC aggregates harvested from bioreactor at days 4 and 8 were dissociated using the protocols described before (section 1.2.2) and inoculated at 2×10^4^ cell/cm^2^ in collagen I coated flasks. Cells were cultured for 4 days in adherent static culture systems according to the standard protocol (section 1.1). For the evaluation of cell expansion capacity, cells were harvested (section 1.1) and the number of total cells was estimated by crystal violet nuclei staining assay (section 1.4.3).

**1.4.5. ALDH activity determination by flow cytometry**

ALDH activity of CSCs was determined using ALDEFLUOR™ Kit assay (Stem Cell Technologies) according to manufacturer’s instructions and analyzed by flow cytometry using a CyFlow® space (Partec GmbH) instrument, registering 30000 events/sample. Data analysis was performed by Flowing Software.

**1.4.6. SFU assay**

Sphere forming unit assay was performed on NSCLC aggregates harvested from bioreactors at days 4 and 8 and dissociated using the protocols described before (section 1.2.2). Cells were seeded in triplicate in ultra-low-attachment 24-well plates (Corning Inc.) (1.5×10^3^cell/well) and cultured in SFM. The number of spheres (3D multicellular structures greater than 50 µm in diameter) was assessed by light microscopy after 5-6 days and up to five passages. Spheres passage was performed by inoculating 1×10^4^cell/mL in ultra-low attachment flasks (Corning Inc) in serum free medium and split using the standard protocol described in section 1.1.

- 1. **Statistical analysis**

Statistical analysis was carried out using GraphPad Prism 5 software. Data are mean ±SD from at least duplicate data. One-way ANOVA analysis with Turkey’s post multiple comparison test was performed to assess differences between cell dissociation protocols. Statistical significance was indicate as follows *P<0.05, **P<0.01, and ***P<0.001.

**2. Supplemental Figures:**


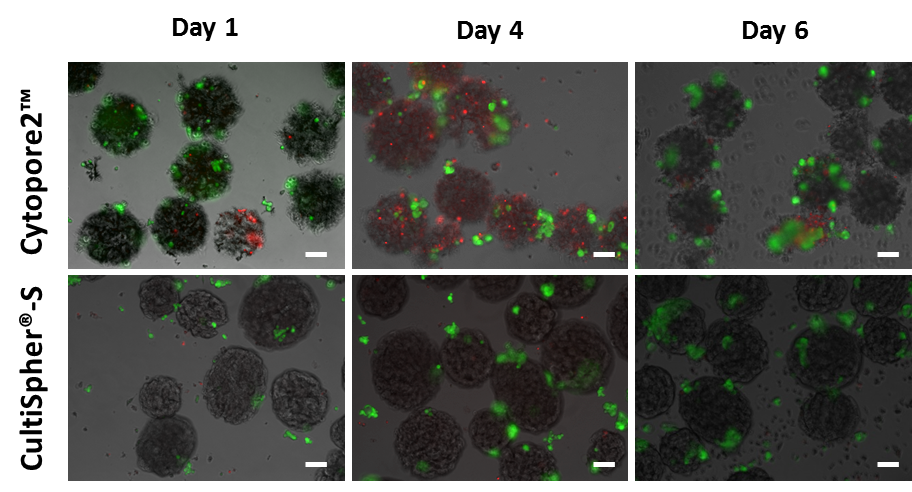


Figure S1: Phase contrast and fluorescence microscopy images of CRC cells cultured on macroporous microcarriers, Cytopore2^TM^ and Cultisphere®-S, at days 1, 4 and 6. Viability analysis of cultures stained with fluoresceine diacetate (FDA – live cells, green) and propidium iodide (PI – dead cells, red). Scale bars: 100 µm.


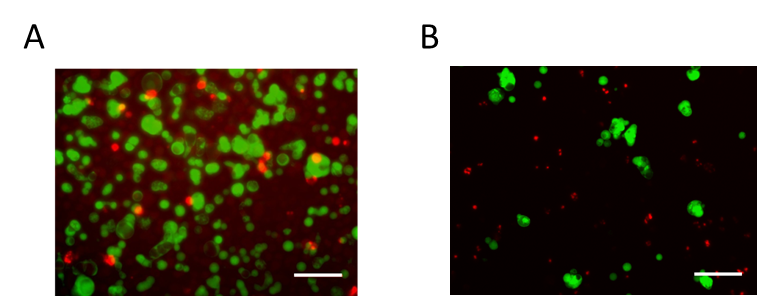


Figure S2: Fluorescence microscopy images of CRC cultures at day 4 on BR-F/T using a culture medium with serum (A) and a serum free medium (B). Viability analysis of cultures stained with fluoresceine diacetate (FDA – live cells, green) and propidium iodide (PI – dead cells, red). Scale bars: 100 µm.


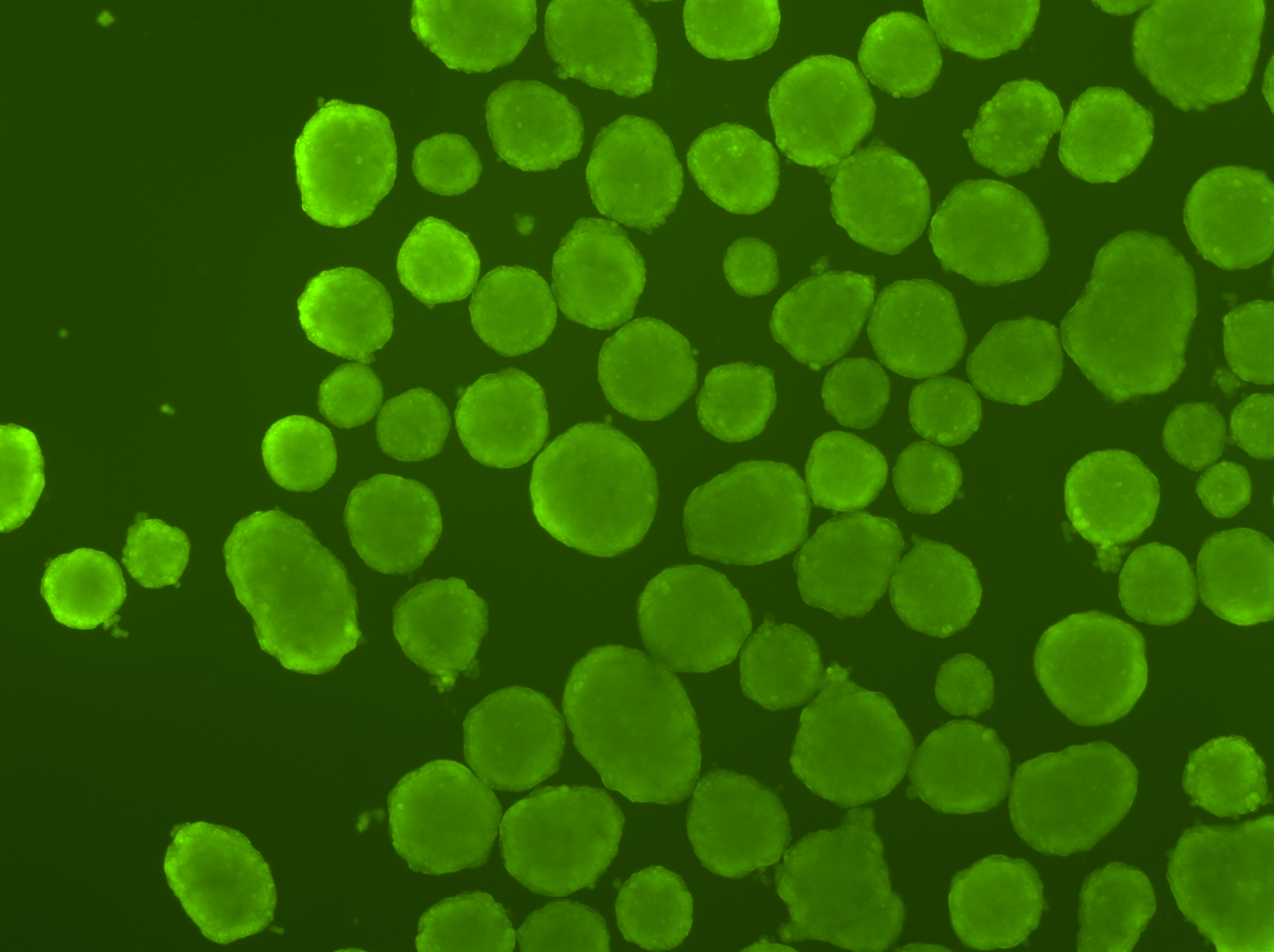

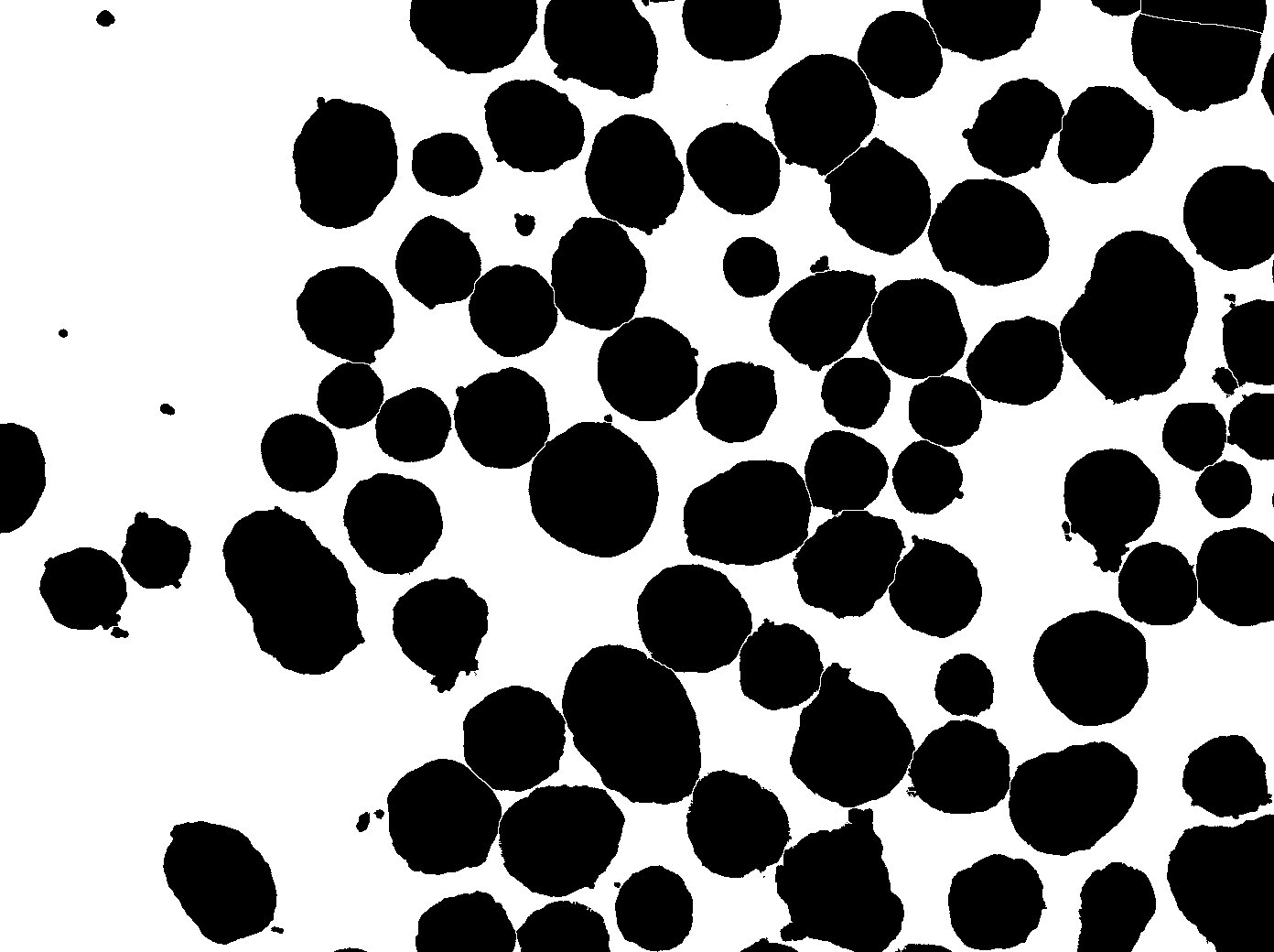

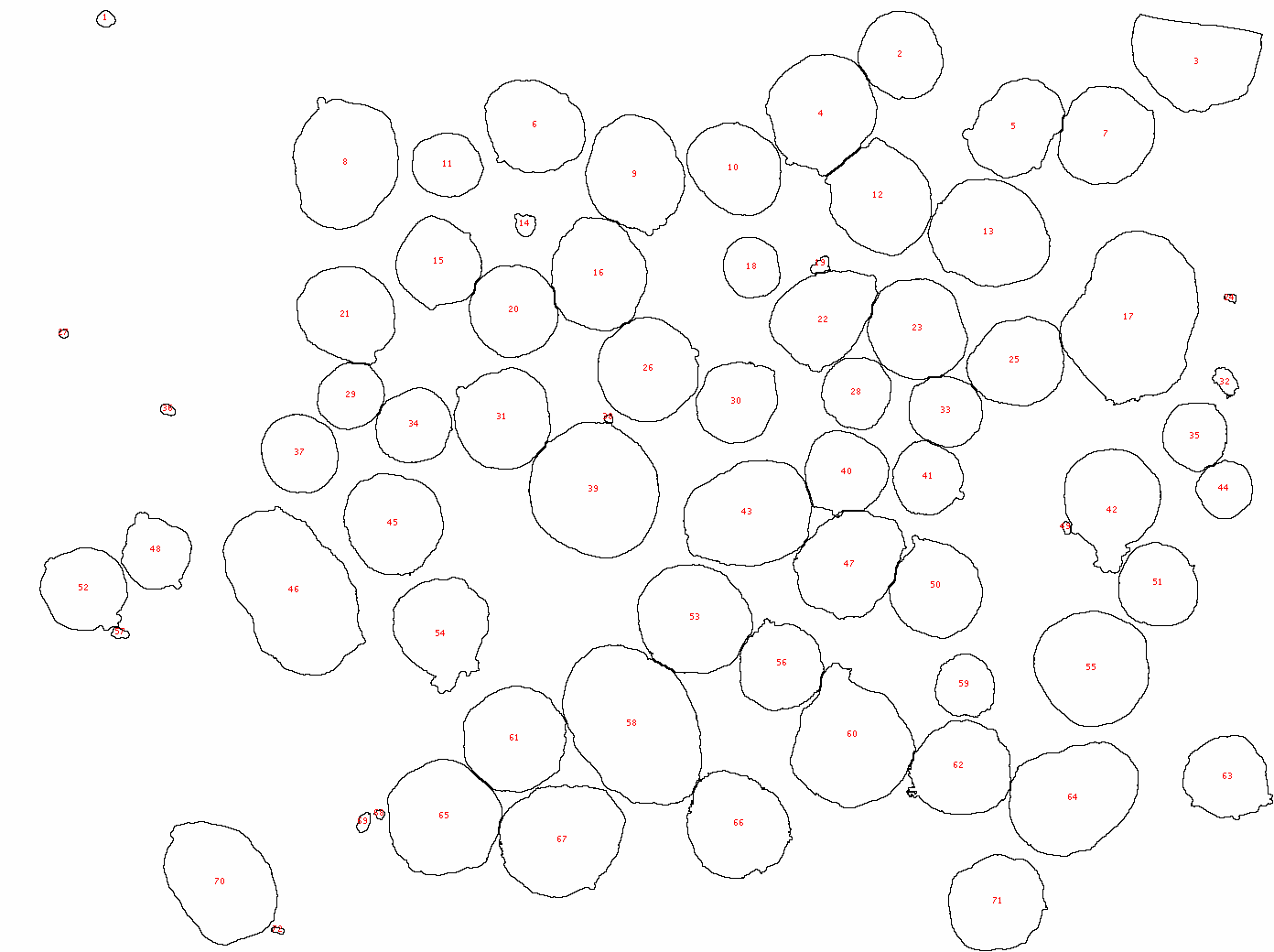


Figure S3: Representative image of the ImageJ mask for aggregate size distribution analysis.

**3. Supplemental Tables:**

**Table S1.** Main characteristics of microcarriers tested. Classification of microcarrier type was indicated according to Chen *et al.* (2013)^11^; (ECM- extracellular matrix)

| **Microcarrier** | **Type** | **Matrix** | **Dimension (pore size)** | **Surface feature** | **Density** |
| --- | --- | --- | --- | --- | --- |
| **Cytodex 1™**  (GE Healthcare) | Positive charged | Dextran | Spherical  Ø 147‐248 μm | Diethylaminoethyl | 1.03 g/cm^3^ |
| **P Plus 102-L**  (Thermo Scientific HyClone) |  | Polystyrene | Spherical  Ø 125‐211 μm | Cationic charged | 1.02 g/cm^3^ |
| **FACT 102-L**  (Thermo Scientific HyClone) |  | Polystyrene | Spherical  Ø 125-212 μm | Cationic charged and type I porcine collagen | 1.02 g/cm^3^ |
| **Cytodex 3™**  (GE Healthcare) | Collagen coated | Dextran | Spherical  Ø 141‐211 μm | Denatured pig skin Type I collagen | 1.04 g/cm^3^ |
| **CGEN 102-L**  (Thermo Scientific HyClone) |  | Polystyrene | Spherical  Ø 125‐212 μm | Type I porcine collagen | 1.02 g/cm^3^ |
| **Pro-F 102-L**  (Thermo Scientific HyClone) | ECM coated | Polystyrene | Spherical  Ø 125‐212 μm | Recombinant fibronectin | 1.02 g/cm^3^ |
| **Cytopore2™**  (GE Healthcare) | Macro-porous | Cotton cellulose | Spherical  Ø ≈230 μm (≈30 μm) | Diethylaminoethyl | 1.03 g/cm^3^ |
| **Cultispher®-S**  (Percell) |  | Gelatin | Spherical  Ø ≈130-380μm (10-20 μm) | Uncoated | 1.04 g/cm^3^ |
